# Supplementary material for: SOX17 regulates uterine epithelial–stromal cross-talk acting via a distal enhancer upstream of Ihh
Source: Nat Commun. 2018 Oct 24;9:4421. doi: 10.1038/s41467-018-06652-w (PMC6200785; doi:10.1038/s41467-018-06652-w)
Supplement: Supplementary file 1 — Supplementary Information [file 41467_2018_6652_MOESM1_ESM.pdf]

Supplementary Information

Wang et al. SOX17 regulates uterine epithelial-stromal crosstalk acting via a distal enhancer upstream of *Ihh*

Contents:

Supplementary Tables 1 and 2
Supplementary Figures 1-4

Supplementary Table 1. Antibody information.

Antibody	Vender	Cat#	Host species	Dilution for IHC
ARID1A	Santa Cruz	sc-98441	Rabbit	1:500
COUP-TFII	R&D Systems	PP-H7147-00	Mouse	1:300
ESR1	Santa Cruz	sc-543	Rabbit	1:200
FOXA2	Cell Signaling	8186	Rabbit	1:400
HAND2	R&D Systems	AF3876	Goat	1:200
Ki67	Abcam	ab15580	Rabbit	1:1000
pESR1	Santa Cruz	sc-101675	Rabbit	1:200
pFRS2	R&D Systems	AF5126	Rabbit	1:100
PGR	Dako	A000098	Rabbit	1:400
PTCH1	Novus Biologicals	NBP1-71662	Rabbit	1:200
PTCH2	LifeSpan BioSciences	LS-B301	Rabbit	1:500
SOX17	R&D Systems	AF1924	Goat	1:500
TRP63	Cell Signaling	13109	Rabbit	1:800
Isolectin B4	Sigma	L2140	N/A	1:200

Supplementary Table 2. Primer information.

Gene symbol	Primer	Vendor
Ihh	Mm00439613_m1	Thermo Fisher
Sox17	Mm00488363_m1	Thermo Fisher
Foxa2	Mm00839704_mH	Thermo Fisher
Lif	Mm00434761_m1	Thermo Fisher
Lifr	Mm00442940_m1	Thermo Fisher
Trp63	Mm00495793_m1	Thermo Fisher
Ltf	Mm00434787_m1	Thermo Fisher
Greb1	Mm00479269_m1	Thermo Fisher
Areg	Mm00437583_m1	Thermo Fisher
Ptch1	Mm00970977_m1	Thermo Fisher
Nr2f2	Mm00772789_m1	Thermo Fisher
Hand2	Mm00439247_m1	Thermo Fisher
Fgf9	Mm00442795_m1	Thermo Fisher
Fgf12	Mm00802587_m1	Thermo Fisher
Foxo1	Mm00490672_m1	Thermo Fisher
Gli1	Mm00494654_m1	Thermo Fisher
Gli2	Mm01293117_m1	Thermo Fisher
Msx1	Mm00440330_m1	Thermo Fisher
Msx2	Mm00442992_m1	Thermo Fisher
Notch1	Mm00435245_m1	Thermo Fisher
Notch3	Mm00435270_m1	Thermo Fisher
Wnt4	Mm01194003_m1	Thermo Fisher

Supplementary Fig. 1

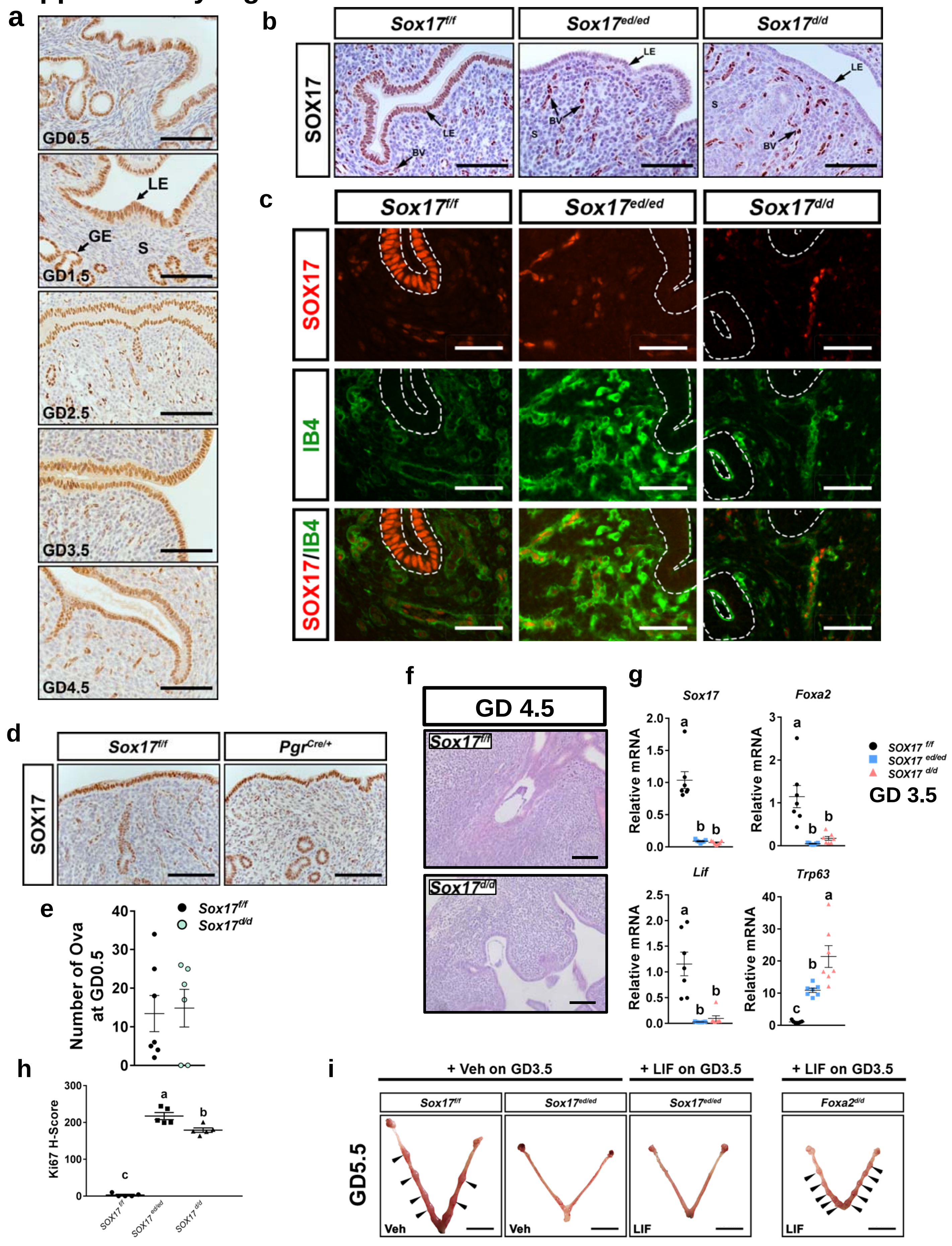

Supplementary Fig. 1 | (related to Fig. 1) Conditional deletion of *Sox17* in the adult mouse uterus. *Sox17*$^{f/f}$ mice were bred with *Ltf*^{iCre} and *Pgr*^{Cre} mice separately, to delete *Sox17* conditionally in adult mice. **a,** Temporal and spatial expression of SOX17 in cross-sections of the adult mouse uterus throughout the preimplantation period, Gestation Day (GD) 0.5 to 4.5 (*n*=3). GE, glandular epithelium; LE, luminal epithelium; S, stroma. Scale bars: 100μm. **b**, Immunolocalization of SOX17 in cross-sections of GD 3.5 uteri from *Sox17*$^{f/f}$ (control), *Sox17*$^{ed/ed}$ (*Ltf*^{iCre}*Sox17*$^{f/f}$), and *Sox17*$^{d/d}$ (*Pgr*^{Cre}*Sox17*$^{f/f}$) female mice (*n*=4). Scale bars: 100μm. **c**, Immunocolocalization of SOX17 (red) and endothelial cell marker, isolectin B4 (green) in cross-sections of GD 3.5 uteri from *Sox17*$^{f/f}$, *Sox17*$^{ed/ed}$ and *Sox17*$^{d/d}$ mice (*n*=3). Scale bars: 50μm. Dotted lines outline uterine epithelia. **d,** Immunohistochemical staining of SOX17 in uteri between *Sox17*$^{f/f}$ and *Pgr*$^{Cre/+}$ mice (*n*=3) at GD3.5. Scale bars: 100μm. **e,** Number of ova ovulated from *Sox17*$^{f/f}$ (black dots, n=7) and *Sox17*$^{d/d}$ (light blue dots, n=6) at GD0.5. **f,** Hematoxylin and eosin stain staining for embryo attachment at GD4.5 (n=3). Scale bars: 100μm. **g,** Quantification of *Sox17, Foxa2, Lif and Trp63 mRNAs* in 2-month old uteri of *Sox17*$^{f/f}$ (black dots), *Sox17*$^{ed/ed}$ (blue squares) and *Sox17*$^{d/d}$ (pink triangles) mice on GD 3.5 (*n*=7). Different superscript letters denote significant (*P*<0.05, ANOVA with Tukey's post-hoc test) differences. Data are presented as means ± S.E.M. **h,** H-score quantification of Ki67 in endometrial section of *Sox17*$^{f/f}$ (black dots), *Sox17*$^{ed/ed}$ (black squares) and *Sox17*$^{d/d}$ (black triangles) mice (*n*=3) at GD 3.5. Different superscript letters denote significant (*P*<0.05, ANOVA with Tukey's post-hoc test) differences. Data are presented as means ± S.E.M. **i,** Embryo implantation sites (IS, indicated by arrow) were observed on GD 5.5 in vehicle-treated *Sox17*$^{f/f}$ mice (n=5) and in LIF-replaced *Pgr*$^{Cre/+}$*Foxa2*$^{f/f}$ (*Foxa2*$^{d/d}$, n=3) mice but neither in vehicle-treated nor LIF-replaced *Sox17*$^{ed/ed}$ mice (*n*=5).

Supplementary Fig. 2

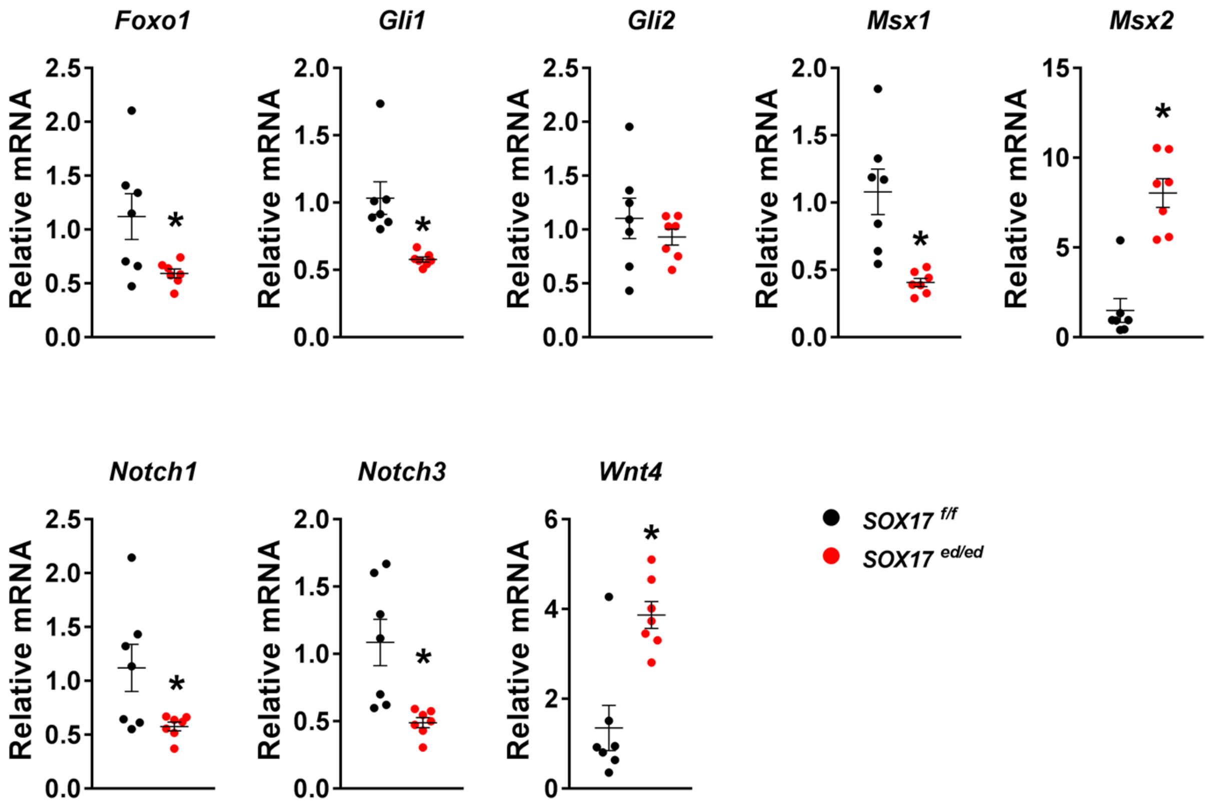

Supplementary Fig. 2 | (related to Fig. 4) Validation of *Sox17* KO microarray by qPCR analysis. Black dots denote *Sox17*$^{f/f}$ group and red dots denotes *Sox17*$^{ed/ed}$ group. *P<0.05 (Student's t test). Data are presented as means ± S.E.M.

Supplementary Fig. 3

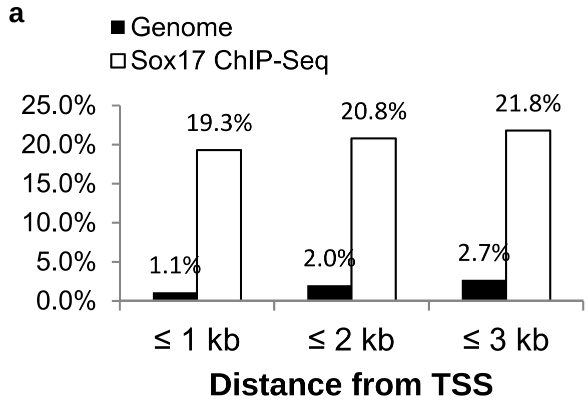

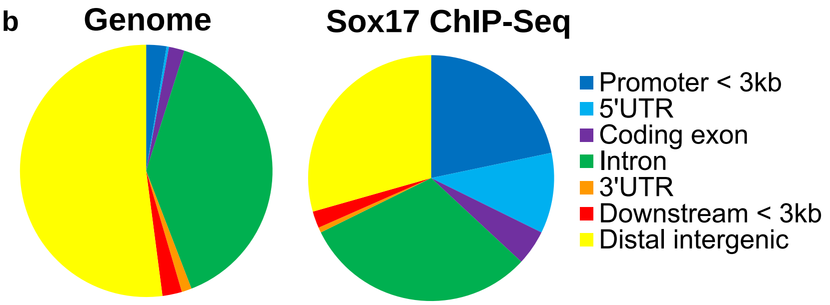

c

Rank	p-value	Motif	Matches to known motifs
1	1.00E-1514	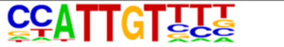	SOX2, SOX3, SOX6, SOX9, SOX10, SOX14, SOX15, SOX30, SRY2
2	1.00E-124	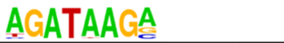	GATA1, GATA2, GATA3, GATA4, GATA6
3	1.00E-118	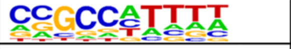	E2F2, E2F3, NANOG, SMAD3, YY1, YY2
4	1.00E-117	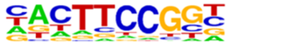	ELF1, ELK1, ELK3, ELK4, ETS, ETV1, FEV, GABPA
5	1.00E-103	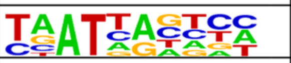	EVX1, EVX2, HOXB2, LHX1, LHX4, LHX8, LHX9, MEOX2, NKX6-2, VAX2
6	1.00E-83	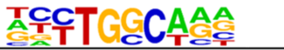	HAND1, HIC1, MEIS1, MEIS2, NF1, NFIA, NFIC, NFIX, TGIF1, TGIF2
7	1.00E-64	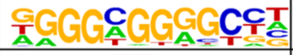	KLF4, KLF5, KLF7, KLF14, MAZ, SP1, SP2
8	1.00E-63	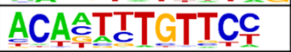	AR, GR, NR3C1, NR3C2, PGR, SOX4
9	1.00E-57	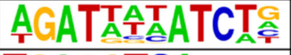	CHOP, CPHX, DUX4, DUXA, GATA2, GATA3, GATA5
10	1.00E-54	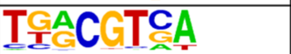	ATF1, ATF7, CRE, CREB1, CREB5, CREM, JUNDM2

d

Wildtype vs. *Ihh19*$^{d/d}$

Chr1:75,018,873-75,018,154; 5'-3'

```
Wildtype     AGGTCACCACTTTTGAGGCTTTTTCAGGAAGCTCAGTTGGAAGCCCTGTCTTCTCTGGGT  480
Ihh19d/d     AGGTCACCACTTTTGAGGCTTTTTCAGGAAGCTCAGTTGGAAGCCCTGTCTTCTCTGG--  355
             **********************************************************

Wildtype     TCCTGTCACCATTGGCTTATAATTCTGTTACAGCAGATCACTTGCCACACAAAACAAAAC  540
Ihh19d/d     ------------------------------------------------------------  355

Wildtype     TGTAAGTGTATGCCTGTCACACACATGCAAATGTGAGTTTTCAGAGGTTAGCTTTCACTG  600
Ihh19d/d     ------------------------------------------------------------  355

Wildtype     ATGGCTGGTCAATGTTTCACTCAGATCTGTTCTCAGGAGGGCAGGCCTGAGGTGTCCACC  660
Ihh19d/d     ------------------------------------------------------------  355

Wildtype     CGCTGCACCTCCTCCTATAGTGCCTGCTATCTATAATCCCAGCCCACCCACCCCCAAGGG  720
Ihh19d/d     ------------------------------------------------------------  355

Wildtype     GGTGGGGGTGAGGCTTGAATTGTCTCTGGGCTAATCAGATGGATGGTCATGCTGTAGGCT  780
Ihh19d/d     ------------------------------------------------------------  355

Wildtype     CACCTGACTCTTCTTGCCCTGCCTGCCGTTGTGGTCAGTGACCCGCTCTATGCTGGTACA  840
Ihh19d/d     ------------------------------------------------------------  355

Wildtype     TGGTGCTCCTGGCTAGGCATACTTACAGCAGGAGACACTATTTACTTTGCTTAGTTCCAC  900
Ihh19d/d     ------------------------------------------------------------  355

Wildtype     TCCAGCATTCTTGCTTTGATCTCTGTGGCTGCCCCACCTCACCCCTGCCATTGTCCTGCC  960
Ihh19d/d     ------------------------------------------------------------  355

Wildtype     CTGGGAGCTCATGCTGTTCCTTCCGTAGCTCAGGTGGCCATACAGCTCTCTTCCCTGGCC  1020
Ihh19d/d     ------------------------------------------------------------  355

Wildtype     ATTCTGGGAGCATGTGCAGGGATGAGTCCTTAAGGGCTCCAGTCTGTACCAGTTGTTGCT  1080
Ihh19d/d     -----------------------------------GCTCCAGTCTGTACCAGTTGTTGCT  380
                                                *************************

Wildtype     TCCCCCCACCCCCACATTCAGTTGCTTTCTTTTTATCTGTTCTGTTTTCTTGTCACCTTA  1140
Ihh19d/d     TCCCCCCACCCCCACATTCAGTTGCTTTCTTTTTATCTGTTCTGTTTTCTTGTCACCTTA  440
             ************************************************************
```

Supplementary Fig. 3 | (related to Fig. 6) a, The percentage of ChIP-seq peak regions that reside in a defined (1, 2, or 3-kb) region upstream of gene boundaries was calculated and compared with the genome background percentages of the same region. **b**, Enrichment distribution of SOX17 binding on the genome compared to normal expected enrichment using the CEAS module at Cistrome. Promoter region was defined in increments of 2.5 kb up to 3 kb. When the SOX17-binding interval is within a gene, it is further defined as within the 5'-untranslated region (UTR), 3'-UTR, coding exon, or intron. Intergenic region is defined as more than 3 kb from gene boundaries. **c**, Listing of top significantly enriched sequence motifs identified using the HOMER *de novo* motif enrichment analysis for the dataset containing SOX17 binding intervals. **d**, Sequence alignment between $Ihh19^{d/d}$ and $Ihh^{+/+}$ (wildtype) mice. Blue and yellow highlight the sequence of gRNA binding sites, and red highlights the sequence of SOX17 binding peaks.

Supplementary Fig. 4

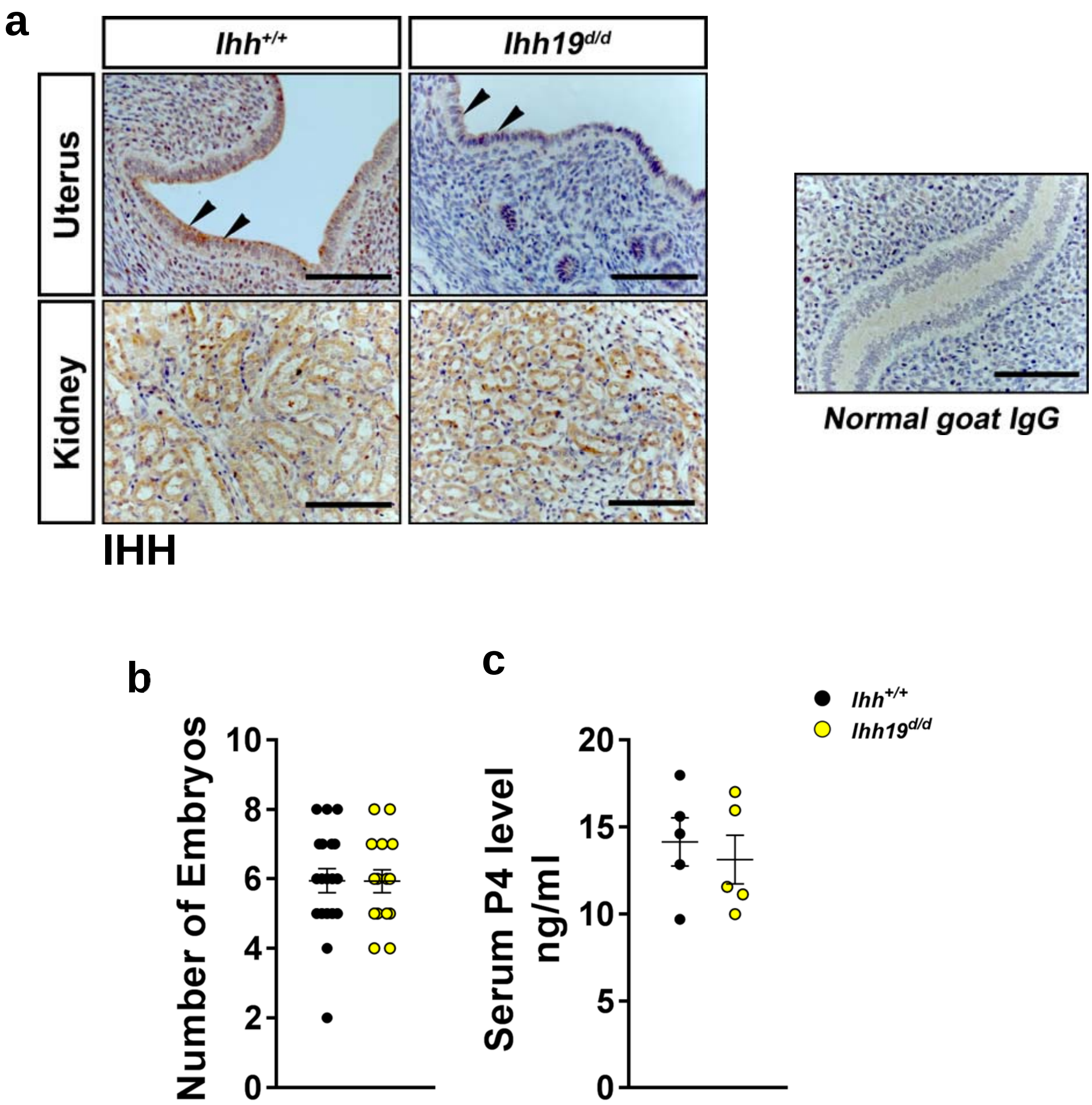

Supplementary Fig. 4 | (related to Fig. 7) a. Immunohistochemical staining of IHH in GD 2.5 uteri and kidney from *Ihh*$^{+/+}$ *and Ihh19*$^{d/d}$ mice (*n*=3). Normal goat IgG served as a negative control. Scale bars, 100µm. **b**. The number of flushed embryo from *Ihh*$^{+/+}$ (black dots) *and Ihh19*$^{d/d}$ (yellow dots) mice at GD2.5 (*n*=15). **c**. P4 level in serum from *Ihh*$^{+/+}$ (black dots) *and Ihh19*$^{d/d}$ (yellow dots) mice at GD2.5 (*n*=5). Data are presented as means ± S.E.M.
