# Supplementary material for: SOX17 regulates uterine epithelial–stromal cross-talk acting via a distal enhancer upstream of Ihh
Source: Nat Commun. 2018 Oct 24;9:4421. doi: 10.1038/s41467-018-06652-w (PMC6200785; doi:10.1038/s41467-018-06652-w)
Supplement: Supplementary file 3 — Description of Additional Supplementary Files [file 41467_2018_6652_MOESM3_ESM.pdf]

Description of Additional Supplementary Files

File Name: **Supplementary Data 1**
Description: List of differentially expressed genes in *Sox17*$^{ed/ed}$ mouse uteri compared with *Sox17*$^{f/f}$ mouse uteri at GD 3.5.

File Name: **Supplementary Data 2**
Description: Functional annotation of altered transcriptome in *Sox17*$^{ed/ed}$ mouse uteri at GD 3.5.

File Name: **Supplementary Data 3**
Description: List of genes commonly regulated in *Sox17*$^{ed/ed}$ and *Foxa2*$^{ed/ed}$ mouse uteri at GD3.5.

File Name: **Supplementary Data 4**
Description: List of genes commonly regulated in *Sox17*$^{ed/ed}$ and *Arid1a*$^{d/d}$ mouse uteri at GD3.5.

File Name: **Supplementary Data 5**
Description: List of genes bound by SOX17 in mouse uteri 6 h after P4.

File Name: **Supplementary Data 6**
Description: SOX17 ChIP-seq binding position.

File Name: **Supplementary Data 7**
Description: List of genes regulated and bound by SOX17 within 25kb upstream and/or downstream of the gene boundary.
